# The soil microbiome increases plant survival and modifies interactions with root endosymbionts in the field

**DOI:** 10.1002/ece3.8283

**Published:** 2022-01-24

**Authors:** Shaniya H. Markalanda, Connor J. McFadden, Steven T. Cassidy, Corlett W. Wood

**Affiliations:** ^1^ Department of Biological Sciences University of Pittsburgh Pittsburgh Pennsylvania USA; ^2^ Present address: Department of Biology University of Florida Gainesville Florida USA; ^3^ Present address: Department of Biology University of Pennsylvania Philadelphia Pennsylvania USA

**Keywords:** field experiment, *Medicago lupulina*, mutualism, parasitism, rhizobia, root‐knot nematodes

## Abstract

Evidence is accumulating that the soil microbiome—the community of microorganisms living in soils—has a major effect on plant traits and fitness. However, most work to date has taken place under controlled laboratory conditions and has not experimentally disentangled the effect of the soil microbiome on plant performance from the effects of key endosymbiotic constituents. As a result, it is difficult to extrapolate from existing data to understand the role of the soil microbiome in natural plant populations. To address this gap, we performed a field experiment using the black medick *Medicago lupulina* to test how the soil microbiome influences plant performance and colonization by two root endosymbionts (the mutualistic nitrogen‐fixing bacteria *Ensifer* spp. and the parasitic root‐knot nematode *Meloidogyne hapla*) under natural conditions. We inoculated all plants with nitrogen‐fixing bacteria and factorially manipulated the soil microbiome and nematode infection. We found that plants grown in microbe‐depleted soil exhibit greater mortality, but that among the survivors, there was no effect of the soil microbiome on plant performance (shoot biomass, root biomass, or shoot‐to‐root ratio). The soil microbiome also impacted parasitic nematode infection and affected colonization by mutualistic nitrogen‐fixing bacteria in a plant genotype‐dependent manner, increasing colonization in some plant genotypes and decreasing it in others. Our results demonstrate the soil microbiome has complex effects on plant–endosymbiont interactions and may be critical for survival under natural conditions.

## Graphical abstract

We show that the soil microbial community affects survival and interactions with two major root endosymbionts (nitrogen‐fixing bacteria and parasitic root‐knot nematodes) in the field in the legume *Medicago lupulina*. Our experiment demonstrates that the significant fitness consequences of host‐associated microbial communities that have been observed in the laboratory persist under natural conditions, and suggests that the role of soil microbial communities in mediating key endosymbioses merits future research.

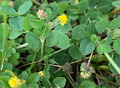

## 1 INTRODUCTION

Plants interact with complex microbial communities comprised of thousands of bacterial and fungal taxa that live in the soil surrounding plant roots (Berendsen et al., 2012; Turner et al., 2013; Vandenkoornhuyse et al., 2015). Plant‐associated soil microbes impact plant traits and plant performance in myriad ways, ranging from producing metabolites that boost plant growth, providing protection against parasites and pathogens, increasing water retention, and improving gas exchange (Busby et al., 2017; Friesen et al., 2011; Panke‐Buisse et al., 2015). Soil microbes impact plant performance indirectly by mineralizing organic matter in the soil, which is important for nutrient cycling and improved soil structure, as well as indirectly by producing biochemicals required by the plant, such as hormones and enzymes (Miransari, 2013). A recent meta‐analysis found that plant‐associated microbes play a major role in ameliorating or buffering plants against abiotic stress (Porter et al., 2020), and can even impact plant geographic range limits (Afkhami et al., 2014).

In addition to these direct effects on plant performance, plant‐associated microorganisms can also significantly change plant interactions with other species, such as herbivores and pollinators, as well as parasites, pathogens, or mutualists (Berg and Koskella 2018; Fox, 1988; Porter et al., 2020; Simonsen & Stinchcombe, 2014). Belowground mutualists ameliorate the negative effects of plant enemies such as pathogens and herbivores by inducing an immune response that confers systemic resistance against a broad range of enemies (Klein et al., 2012; Morris et al., 2010; O’Brien, 2019; Pieterse et al., 2014). The effect of the soil microbiome on plant species interactions can be surprisingly broad: Recently, Hubbard et al. (2019) found that the root microbiome played a larger role in mediating the damage caused by herbivores than plant genotype (Hubbard et al., 2019).

However, our current understanding of the soil microbial community's impact on plant traits and fitness remains limited for two key reasons. First, most soil microbiome studies have been performed under highly controlled conditions in the laboratory or greenhouse (Forero et al., 2019; Petipas et al., 2021). By contrast, relatively few experiments have manipulated plant‐associated microbial communities under natural or semi‐natural conditions (but see Morris et al., 2010; Petipas et al., 2020; Simonsen & Stinchcombe, 2014). The lack of field‐based manipulations of soil microbial communities is a crucial gap in the current literature because experiments across systems have repeatedly found that processes documented under greenhouse conditions are not necessarily reflective of what happens in the field (De Long et al., 2019; Forero et al., 2019; Schittko et al., 2016). Under natural conditions, plants and their associated microbial communities experience substantial environmental variation, as well as abiotic and biotic stresses, all of which can impact the composition and function of soil microbes and ultimately, how they interact with the host (Brown et al., 2020; Zhalnina et al., 2015). Elevated environmental variability and stress in the field relative to the laboratory could either exacerbate or mitigate the microbiome's effect on the plant host (Petipas et al., 2020). Therefore, field experiments are crucial to accurately characterize how soil microbial communities impact plants in their natural habitats.

Second, relatively few studies have considered how the soil microbial community impacts the intimate association plants form with belowground endosymbionts. Throughout this manuscript, we use the term “symbiosis” in its broadest sense to refer to host‐associated microorganisms regardless of their effect on host fitness. These endosymbionts often have major effects on plant fitness. While some are beneficial (e.g., mutualistic mycorrhizal fungi and nitrogen‐fixing rhizobia bacteria), many others are parasites or pathogens (e.g., root‐knot nematodes, fungal pathogens) that form permanent feeding structures in host tissues, stealing resources, and stunting plant growth or increasing mortality (Bird, 1974; Bonkowski, 2004; Friesen et al., 2011; Grman et al., 2012; Masson‐Boivin & Sachs, 2018; O’Keeffe et al., 2021). Determining how the entire soil microbial community impacts the formation and function of widespread endosymbioses with major effects on host fitness is crucial to assess whether microbiome‐mediated effects on host traits fitness are caused by the entire microbiome, impacts on a small number of intimate endosymbioses, or an interaction between the two.

Here, we performed a field‐based microbiome manipulation in the legume *Medicago lupulina* to test how the soil microbiome impacts plant traits, performance, and interactions with two root endosymbionts under field conditions. We exposed field‐grown plants to intact and depleted soil microbiome treatments using sterile and live soil inoculum. In a fully factorial design, we crossed this microbiome manipulation with a manipulation of a major belowground parasite, the root‐knot nematode *Meloidogyne hapla*. Finally, we controlled plants’ exposure to a major belowground mutualist, the mutualistic nitrogen‐fixing bacteria *Ensifer*, by inoculating all experimental plants with a fixed dose of a single rhizobia strain. Because we independently manipulated the entire soil microbiome and these two symbionts, our experiment is able to distinguish between the effect of the entire microbiome on plant performance and the effect of these two major plant endosymbionts. We asked three questions: (1) How does an intact soil microbiome affect plant survival and performance (biomass)?; (2) Does the microbiome affect colonization by parasitic nematodes and nitrogen‐fixing bacteria?; and (3) Does the microbiome modify the cost and benefit of the rhizobia mutualism and nematode parasitism, respectively? We predicted that an intact microbiome would increase plant survival and performance and provide protection against parasite infection.

## 2 METHODS

### 2.1 Study system

**FIGURE 1:**
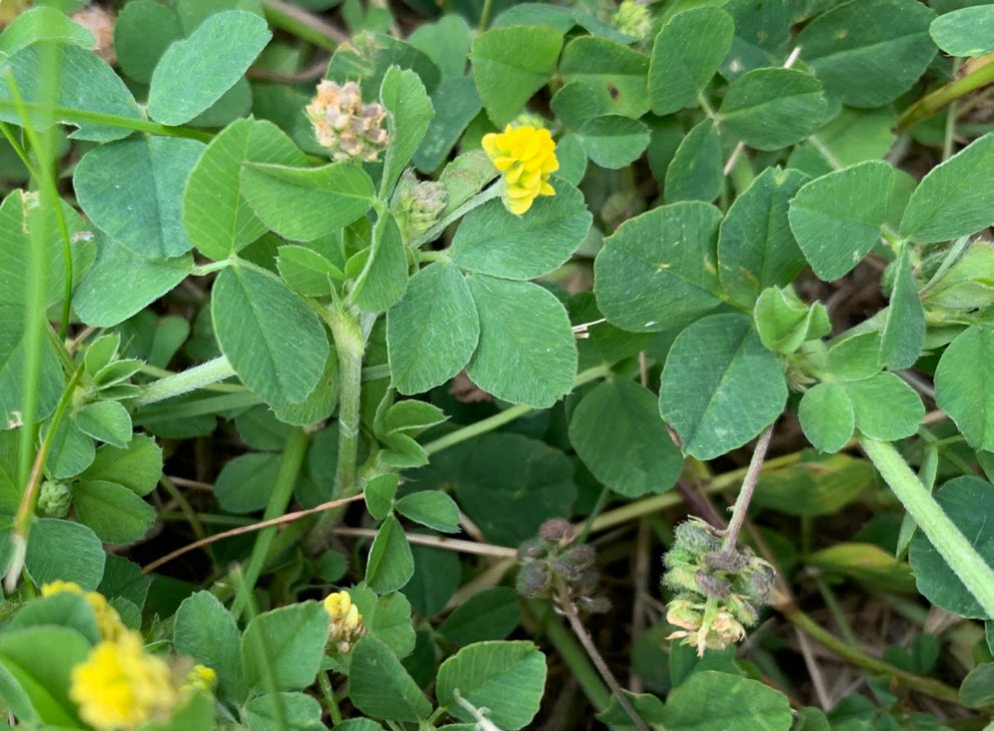
Black medic (*Medicago lupulina*) at our field site at the Pymatuning Laboratory of Ecology in northwestern Pennsylvania. Photo by Shaniya Markalanda

*Medicago lupulina* (Figure 1), the black medick, is an annual or short‐lived selfing perennial weed native to Eurasia (Turkington & Cavers, 1979). It was introduced to North America in the 1700s and is naturalized in disturbed habitats (Turkington & Cavers, 1979). In this study, we focused on two of *Medicago lupulina's* major symbionts: the mutualistic nitrogen‐fixing bacteria commonly known as rhizobia and parasitic root‐knot nematodes. Rhizobia fix atmospheric nitrogen in symbiotic organs called nodules and receive carbon in return (Reeve et al., 2010; Spaink, 2000). They are highly beneficial for their host (Harrison et al., 2017; Vessey, 2003). *Medicago lupulina* interacts with rhizobia in the genus *Ensifer* (formerly *Sinorhizobium*; Young, 2010) (Harrison, Wood, Borges, et al., 2017; Harrison et al., 2017). The northern root‐knot nematode (*Meloidogyne hapla*) is a root parasite that invades the plant and forms gall‐like structures on plant roots (Ali et al., 2017; Bird, 1974; Castagnone‐Sereno et al., 2013). *Meloidogyne hapla* is a generalist parasite that affects many crops worldwide (Jones et al., 2013). Collectively, plant–parasitic nematodes—*Meloidogyne*, cyst‐forming nematodes, and root‐lesion nematodes—are estimated to cause at least $80 billion US dollars in economic losses annually (Jones et al., 2013; Nicol et al., 2011). *Meloidogyne hapla* is common at our field site in northwestern Pennsylvania (C. Wood, personal observation).

The soil microbial community associated with *Medicago* is diverse but dominated by rhizobia: In *Medicago truncatula*, rhizobia are the major bacterial taxa in the nodules, rhizosphere, and root (Brown et al., 2020). Furthermore, the composition of the root endosphere microbial community differs among genotypes, indicating that the host plays some active role in structuring its root‐associated microbial community (Brown et al., 2020). Past research suggests that the microbiome may provide partial protection against parasitic nematodes in plants: Some soil microbes, such as Streptomyces, have been shown to reduce infection by *Meloidogyne* nematodes in tomato plants (Dicklow et al., 1993). The soil microbial community has also been shown to promote nodulation and nitrogen fixation by mutualistic rhizobia in multiple legume species, including the common bean (*Phaseolus vulgaris*) and rooibos (*Aspalanthus linearis*) (Miao et al., 2018; Ramoneda et al., 2021).

### 2.2 Field experiment

**FIGURE 2:**
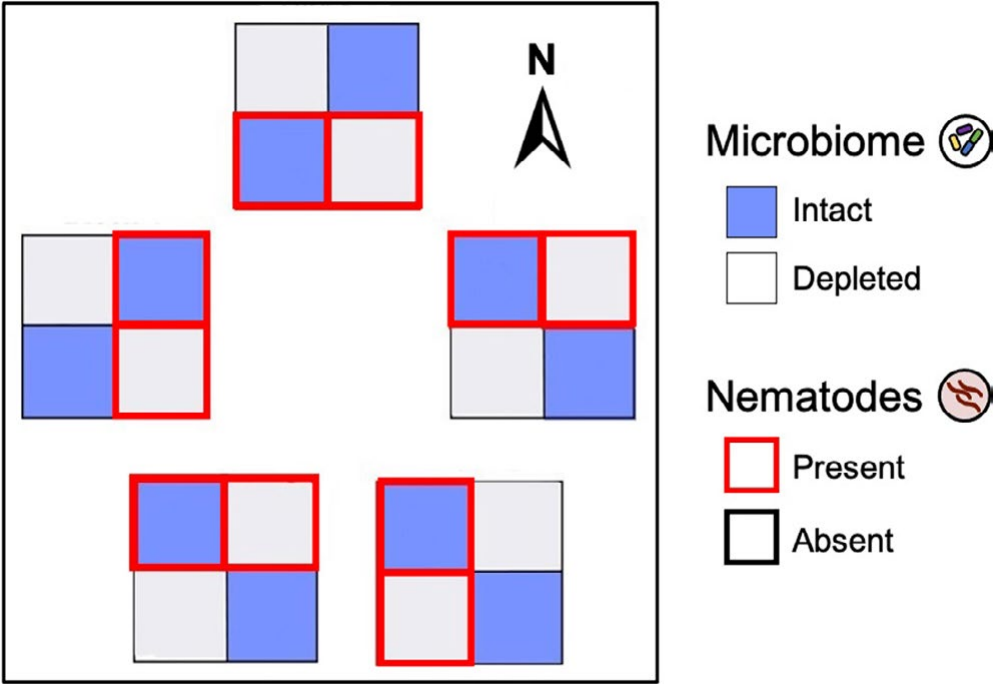
Experimental setup. The experiment consisted of 5 blocks, each of which contained 4 mesocosms (blue and white squares): one per microbiome‐nematode treatment combination. Blue squares represent the intact soil microbiome treatment, while off‐white squares represent the microbe‐depleted soil treatment. A red outline denotes that nematodes were added. All plants were inoculated with rhizobia. Each mesocosm contained six plants (24 plants per block × 5 blocks = 120 plants total)

We performed a field experiment with *M*. *lupulina* to study the impact of the soil microbial community (hereafter, “soil microbiome” or “microbiome”) on plant traits, performance, and belowground interactions with mutualistic rhizobia and parasitic nematodes. We factorially manipulated soil microbiome and nematode presence for a total of four treatments: intact microbiome with nematodes, intact microbiome without nematodes, microbiome‐depleted with nematodes, and microbe‐depleted without nematodes (Figure 2). We inoculated all experimental plants with a single strain of rhizobia because *M*. *lupulina* plants without rhizobia perform extremely poorly (Harrison, Wood, Borges, et al., 2017).

We set up the experiment in a complete block design (Figure 2) in mesocosms in a field at the Pymatuning Laboratory of Ecology's Donald S. Wood Lab (41.6432°N, 80.4279°W). Five blocks were set up in a 300‐m^2^ field plot, with each block containing four mesocosms, one for each of the four treatments, spaced 0.5 m apart. Each mesocosm consisted of a plastic bin (25.4 cm × 25.4 cm × 16.5 cm) with 2 cm of gravel at the bottom. They were sunk 5–6" deep into the soil to buffer against temperature changes and filled to the brim with sand. Holes were drilled into the bottom of each bin to drain excess water. To minimize contamination from surrounding field soil, each bin was covered with shade cloth and nested in a second bin that also contained about 2 cm of gravel to collect runoff water. Water in the nested bins was emptied every few weeks or after a heavy rain. It is important to note that our mesocosms buffered plants from natural soil conditions (e.g., water content, nutrient flux, temperature), so while our design captured natural field conditions aboveground, it did not recapitulate field conditions belowground.

Each mesocosm contained six plants of five different maternal families (hereafter, genotypes)—one of each and a sixth randomly chosen duplicate—for a total of 120 plants (5 blocks × 4 treatments × 6 plants per treatment per block). The five genotypes (PLE‐01‐03, PLE‐02‐07, PLE‐03‐07, PLE‐04‐10, and PLE‐11‐04) were each collected from a different *M*. *lupulina* population separated by 2–15 km around Linesville, Pennsylvania, in the summer of 2018. We originally collected seeds from up to 10 maternal plants per site and haphazardly chose seeds from one maternal plant per site for this experiment. We grew field‐collected seeds in the greenhouses at the University of Pittsburgh for one generation to minimize maternal effects. The plants used in this experiment were the first‐generation offspring of these greenhouse‐grown plants. Seeds were germinated by scarifying using a razor blade, sterilized in bleach and ethanol, plated onto 1% water agar plates, and maintained in the dark at 4°C for a week (Garcia et al., 2006). The seeds were then planted into sterile sand in plug trays and maintained under ambient conditions (on the windowsill of our field laboratory) and top‐watered for a month before transplantation into field mesocosms.

Month‐old seedlings were transplanted into 1.5″ × 8.25″ Cone‐tainer pots made from autoclavable polypropylene (#SC10R, Stuewe and Sons, Tangent, OR) containing either a live sand‐soil mixture (intact microbiome treatment) or the same sand‐soil mixture that we sterilized by autoclaving it twice at 121°C for 60 min each time (microbiome‐depleted treatment). For our soil inoculum, we sampled soil from our field site at the Pymatuning Laboratory of Ecology (41.6432°N, 80.4279°W). Soil was collected from within 10cm of the root systems of wild *M*. *lupulina* plants. The collected soil was sieved through a large sieve that removed large soil invertebrates but did not remove nematodes and then mixed with sand in a ratio of 10% inoculum to 90% sand (volume/volume). We planted into Cone‐tainers within each mesocosm to prevent belowground competition between experimental plants in the same mesocosm.

Upon transplantation into the field mesocosms, we inoculated all experimental plants with rhizobia and inoculated half the plants with root‐knot nematodes at the same time. Nematodes were collected from *M*. *lupulina* plants harvested near our field site (41.569 N, −80.457 W). We determined that they were the northern root‐knot nematode *Meloidogyne hapla* based on gall morphology. To isolate *M*. *hapla* eggs from the galls of infected plants, we used a bleach extraction protocol: We washed the roots with tap water to remove soil and debris, vigorously shook the roots in 10% commercial bleach for 5 min, and then poured the bleach‐root mixture through sieves, using the #500 sieve to catch the eggs (Eisenback, 2000). We then washed the eggs to remove the bleach, resuspended the eggs in water, and inoculated each plant with ~300 eggs.

Rhizobia were cultured from a nodule of M. lupulina collected near our field site. We sterilized the field‐collected nodules in 95% ethanol and bleach for 20 s, crushed the nodules, and plated the strains onto a 2% tryptone yeast (TY) plate (Harrison, Wood, Borges, et al., 2017). These strains were then restreaked onto TY agar and grown at 30°C for 48 h before being transferred to liquid TY media and cultured for another 48 h at 29°C. These liquid cultures were diluted to an OD600 of 0.1; then, 1mL of this inoculum was added to each plant (Simonsen & Stinchcombe, 2014).

Plants that died during the first four days of the experiment were replaced; plants that died after that were considered “dead” for later analyses. Any replaced plants were replaced with the same genotype unless we ran out of seedlings for that genotype, at which point they were replaced by a haphazardly selected genotype.

### 2.3 Data collection

Plants were harvested 12 weeks after the start of the experiment (July 6 to September 26, 2019). The shoots were placed into individual brown paper bags and dried in a drying oven before being weighed to the nearest 0.0001 g. The roots were rinsed and stored in plastic bags at 4°C, to preserve them for counting nodules and galls, to quantify colonization by rhizobia and nematodes. Gall and nodule structures on the roots were counted under a dissecting microscope. Galls formed by parasitic nematodes and nodules formed by mutualistic rhizobia are clearly visible under a dissecting scope and can be counted to quantify the level of nematode infection and rhizobia colonization on the host plant (Wood et al., 2018). After galls and nodules were counted, roots were dried in a drying oven and weighed to the nearest 0.001 g.

### 2.4 Statistical analysis

We fit all (generalized) linear mixed models in R version 3.5.1 (R Core Team, 2018) using the glmmTMB package (Brooks et al., 2017) unless otherwise noted. These models were fit by maximum‐likelihood estimation, which is robust to unbalanced designs (i.e., unequal sample sizes across treatments) (Bolker et al., 2009). All models included microbiome treatment, nematode treatment, and their interaction as fixed effects. Unless otherwise noted, we fit genotype and block as random effects. We used type III sums of squares and sum‐to‐zero contrasts (Fox & Weisberg, 2019). Following the recommendations of Bolker et al., 2009, we tested significance of fixed effects using Wald chi‐squared tests (executed in the *Anova* function in the car package; Fox & Weisberg, 2019) and tested significance of random effects with likelihood ratio tests. We confirmed that residual error was normally distributed, homoscedastic, and not overdispersed using the DHARMa package (Hartig, 2018). We performed post hoc Tukey tests and extracted least‐squares means and confidence intervals using emmeans (Lenth, 2020) and created all our data figures in ggplot2 (Wickham, 2016).

To test whether the microbiome or nematode treatments affected survival, we ran a model with survival as the dependent variable (error distribution: binomial). We used the “glm” function for this analysis. We fit genotype and block as fixed because the model would not converge when these terms were fit as random effects. Because our microbiome treatment had such a strong effect on plant survival (see Section 3), we performed all subsequent analyses in two ways: including all plants in the experiment, and only including plants that were alive when harvested. The results were qualitatively similar, so we present only the results for plants that were alive when harvested. Although this treatment‐specific mortality reduced our sample size for subsequent analyses, we still had >10 surviving plants in each treatment combination (M‐N‐: 11 plants, M‐N+: 19, M+N‐: 30, M+N+: 30), sufficient for our most complex model, included 7 fixed effects (7 parameters) and 2 random effects (two additional parameters; Bolker et al., 2009).

There were two plants that were outliers with respect to gall number, which were 18 and 31 standard deviations above the mean gall number of their respective treatment groups. To determine whether these outliers were driving any of the patterns we observed, we ran any models that included gall number with and without these individuals. Their exclusion did not qualitatively affect our results, so below we report the models with the two outliers removed.

To test whether the microbiome or nematode treatments affected plant biomass, we ran three models with shoot biomass, root biomass, and shoot‐to‐root ratio as the dependent variables (error distribution: Gaussian). To test our treatments affected belowground interactions with rhizobia and nematodes, we ran two models with gall number and nodule number as the dependent variables (error distribution: negative binomial, "nbinom2" in R). In these models, we allowed the effect of genotype to vary across microbiome and nematode treatments. We included root biomass as a fixed effect to adjust for differences in overall plant size, but our results were qualitatively similar in models without root biomass as well. We were unable to fully eliminate heteroscedasticity from the nodule number model, so our P‐values for this analysis may be slightly anticonservative.

Finally, we tested whether the microbiome or nematode treatments modified the fitness benefits of the rhizobia mutualism or the costs of nematode parasitism. We tested this hypothesis in a model (error distribution: Gaussian) with shoot mass as the dependent variable. In addition to the microbiome and nematode treatments, this model included nodule number, gall number, and the following two‐way interactions: microbiome‐by‐nodule number, microbiome‐by‐gall number, and nematode‐by‐nodule number. These interaction terms test whether the microbiome or nematode treatments influenced the relationship between nodule or gall number and shoot biomass. We rescaled nodule and gall numbers to z‐scores (mean of 0 and variance of 1) because the model would not converge with raw counts (Bolker et al., 2009).

## 3 RESULTS

**FIGURE 3:**
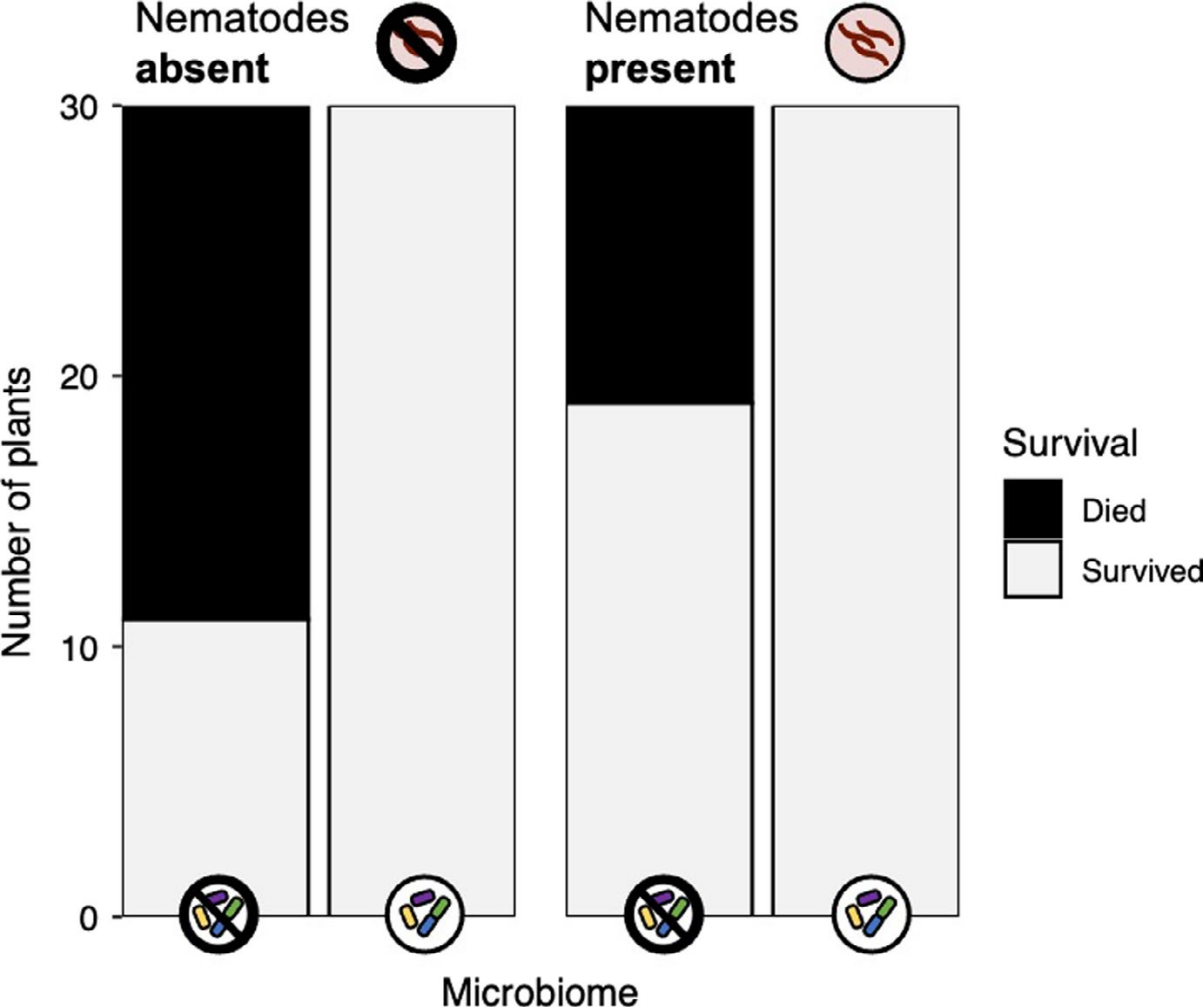
The microbiome, but not parasitic nematodes, affected survival in *M*. *lupulina*. Left: Nematodes absent. Right: Nematodes present. Plants in the microbiome‐depleted treatment were half as likely to survive as plants in the intact microbiome treatment. See Table 1 for statistics

The microbiome treatment impacted plant survival (Table 1, Figure 3). Only 50% of plants in the microbiome‐depleted treatment survived, while 100% of plants in the intact microbiome treatment survived (microbiome main effect: *p* < .001). Nematode infection did not impact survival (nematode main effect: *p* = .999), nor did the effect of the microbiome on survival differ across nematode treatments (microbiome‐by‐nematode interaction: *p* = .999). Plant genotypes differed in their probability of survival (genotype main effect: *p* = .012). Survival also differed among experimental blocks (*p* = .024), indicating that variation in the microenvironmental conditions across our field site contributed to mortality.

**TABLE 1:** The effect of the experimental treatments on survival, shoot mass, root mass, and shoot‐to‐root ratio

	Survival			Shoot mass			Root mass			Shoot:root ratio		
	χ^2^	*df*	*p*	χ^2^	*df*	*p*	χ^2^	*df*	*p*	χ^2^	*df*	*p*
Microbiome	**58.413**	**1**	**<.001**	2.985	1	.084	0.199	1	.656	1.210	1	.271
Nematode	0.000	1	.999	0.043	1	.835	3.019	1	.082	2.631	1	.105
Microbiome × Nematode	0.000	1	.999	0.829	1	.363	1.004	1	.316	1.299	1	.254
Genotype	**12.892**	**4**	.**012**	0.000	1	1.000	0.000	1	1.000	0.468	1	.494
Block	**11.281**	**4**	.**024**	1.014	1	.314	0.563	1	.453	0.000	1	1.000

Note

Genotype and block were fit as fixed effects in the survival model, and as random effects in the other three. We tested significance for fixed effects and random effects with Wald chi‐squared tests and likelihood ratio tests, respectively. Bold text indicates statistically significant terms at *α* = 0.05.

**FIGURE 4:**
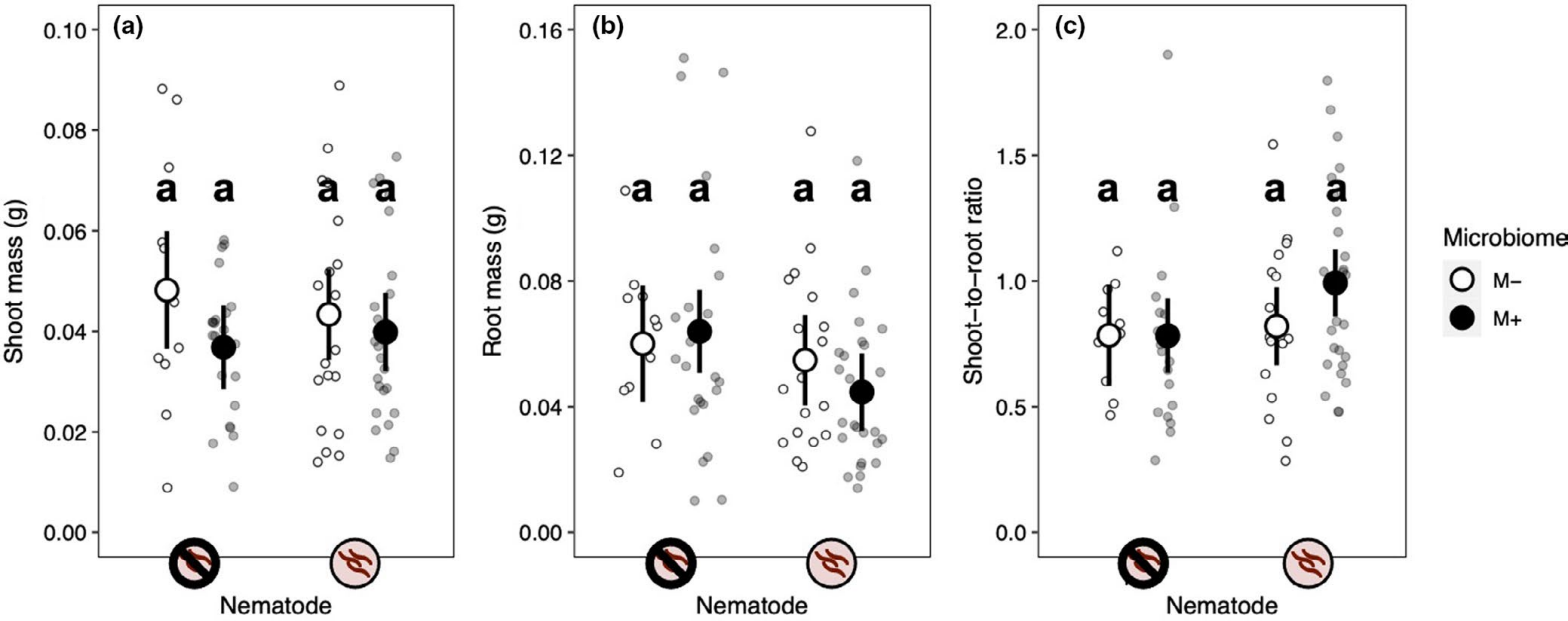
Neither the microbiome nor nematode treatments significantly affected plant performance. (a) Shoot mass. (b) Root mass. (c) Shoot‐to‐root ratio. Small points are the raw data; large points are least‐squares means and 95% confidence intervals. See Table 1 for statistics. Lower‐case letters above the treatment means indicate the results of post hoc Tukey tests (*α* = 0.05)

Neither nematode infection nor microbiome treatment significantly affected shoot biomass, root biomass, or shoot‐to‐root ratio (Table 1, Figure 4). Neither treatment significantly affected shoot biomass (nematode main effect: *p* = .835; microbiome main effect: *p* = .084) (Figure 4a). Neither treatment impacted root biomass (nematode main effect: *p* = .083; microbiome main effect: *p* = .656) (Figure 4b). Neither treatment affected shoot‐to‐root ratio (Figure 4c) (nematode main effect: *p* = .105; microbiome main effect: *p* = .271). The effect of the microbiome treatment on the three traits did not vary across nematode treatments (microbiome‐by‐nematode interactions; Table 1), and plant genotypes did not differ significantly in any of the measured traits (Table 1).

**FIGURE 5:**
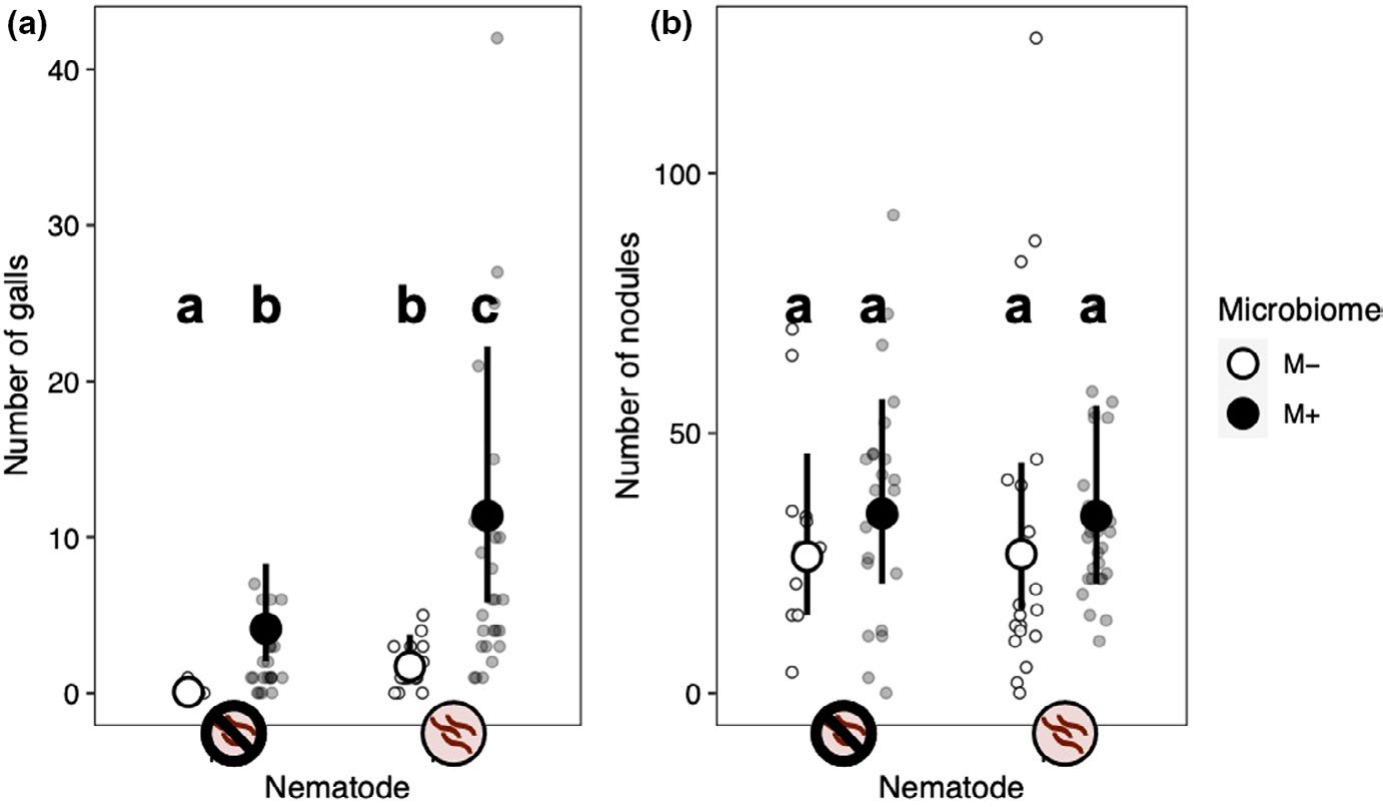
Effect of the nematode and microbiome treatments on (a) gall number and (b) nodule number. Small points are the raw data; large points are least‐squares means and 95% confidence intervals. See Table 2 for statistics. Lower‐case letters above the treatment means indicate the results of post hoc Tukey tests (*α* = 0.05)

**FIGURE 6:**
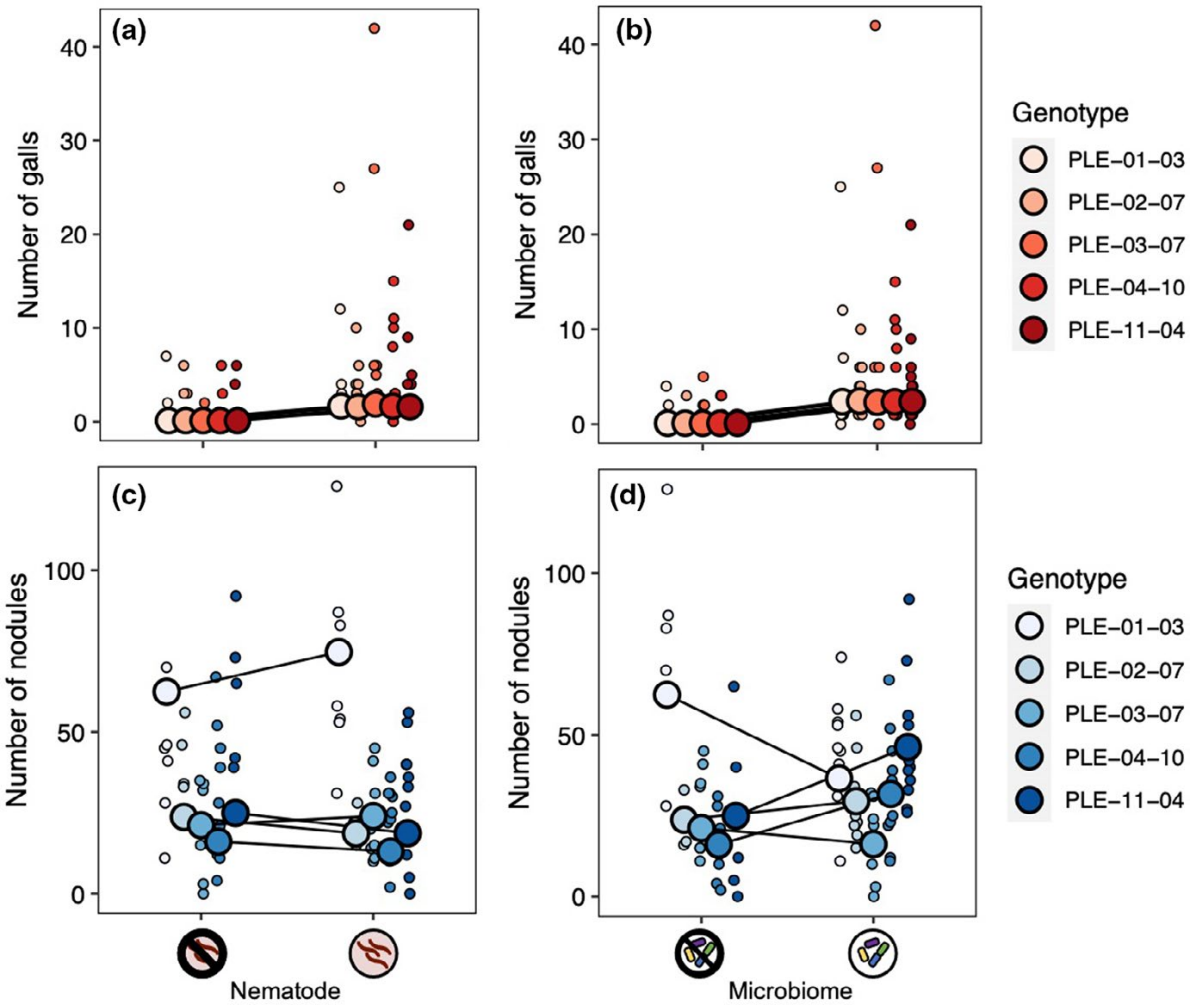
Variation among genotypes in the effect of the nematode treatment (a, c) and microbiome treatment (b, d) on plant endosymbionts. (a) and (b): The number of galls formed by parasitic nematodes. (c) and (d): The number of nodules formed by mutualistic rhizobia. Genotype names refer to the region (PLE: Pymatuning Laboratory of Ecology), population ID, and maternal plant ID from which the seeds were sourced. Small points are the raw data; large points are conditional modes for the genotype random effect, extracted from our models using the function *ggpredict* in the ggeffects package (Ludecke, 2018). See Table 2 for statistics

Both our nematode and microbiome treatments affected gall formation (Table 2, Figure 5a). The main effect of nematode treatment indicates that our nematode inoculation worked well (nematode main effect: *p* < .001). Plants inoculated with nematodes formed 3.9 times more galls than those that were not. The intact microbiome treatment also significantly increased gall formation: Plants with an intact microbiome had 5.5 times more galls than those in the microbiome‐depleted treatment. The effect of the microbiome treatment on gall formation did not vary across nematode treatments (microbiome‐by‐nematode interaction: *p* = .135). Plant genotypes did not differ in gall formation, indicating that genotypes did not differ in resistance to parasitic nematodes, nor did genotypes vary in their response to the microbiome or nematode treatments (Table 2, Figure 6a,b).

**TABLE 2:** The effect of the experimental treatments on gall and nodule number

	Gall number			Nodule number		
	χ^2^	*df*	*p*	χ^2^	*df*	*p*
Microbiome	**19.890**	**1**	**<.001**	0.771	1	.380
Nematode	**13.920**	**1**	**<.001**	0.002	1	.964
Microbiome × Nematode	2.233	1	.135	0.652	1	.419
Root mass	2.239	1	.127	**5.576**	**1**	.**019**
Genotype	0.000	1	1.000	**16.153**	**1**	**<.001**
Genotype:Microbiome	0.000	1	.999	**11.261**	**1**	.**001**
Genotype:Nematode	0.163	1	.686	1.567	1	.211
Block	0.000	1	.999	**14.893**	**1**	**<.001**

Note

Genotype, genotype:microbiome, genotype:nematode, and block were fit as random effects in both analyses. We tested significance for fixed effects and random effects with Wald chi‐squared tests and likelihood ratio tests, respectively. Two outliers were excluded from the gall number analysis. Bold text indicates statistically significant terms at α = 0.05. "Genotype:Microbiome" and "Genotype:Nematode" test whether genotypes varied in their response to the microbiome or nematode treatments, respectively.

Neither nematode infection nor microbiome treatment significantly impacted nodulation (Table 2, Figure 5b). The effect of the microbiome treatment on nodule formation did not differ across nematode treatments (microbiome‐by‐nematode interaction: *p* = .419). Plant genotypes differed in the number of nodules they formed (genotype random effect: *p* < .001; Figure 6c,d), indicative of standing genetic variation for the rhizobia mutualism in *M*. *lupulina*. In addition, there was a significant genotype‐by‐microbiome interaction (*p* = .001), indicating that the effect of the microbiome treatment on nodule formation depended on plant genotype (Figure 6d). An intact microbiome increased nodulation in two plant genotypes (PLE‐04‐10 and PLE‐11‐04), decreased nodulation in a third (PLE‐01‐03), and did not seem to affect nodulation in the remaining two (PLE‐02‐07 and PLE‐03‐07). There was no significant genotype‐by‐nematode interaction, indicating that nodulation in all plant genotypes responded similarly to the nematode treatment (*p* = .211; Figure 6c).

Neither the microbiome nor nematode treatments modified the fitness benefits of the rhizobia mutualism (nodule‐by‐microbiome and nodule‐by‐nematode interactions; Table 3). Plants that formed more nodules had larger shoots regardless of microbiome treatment (nodule main effect: *p* = .008). The microbiome treatment did not change the fitness costs of nematode infection either (gall‐by‐microbiome interaction: *p* = .470). Gall number did not affect shoot mass in either microbiome treatment (gall main effect: *p* = .294).

**TABLE 3:** The experimental treatments did not modify the fitness benefits of nodule formation or the fitness costs of gall formation

	Shoot mass		
	χ^2^	*df*	*p*
Nodule number	**6.861**	**1**	.**009**
Gall number	1.102	1	.294
Microbiome	3.311	1	.069
Nematode	0.706	1	.401
Nodule num. × Microbiome	0.085	1	.770
Gall num × Microbiome	0.521	1	.470
Nodule num. × Nematode	0.130	1	.719
Genotype	0.000	1	1.000
Block	0.350	1	.554

Note

Genotype and block were fit as random effects. We tested significance for fixed effects and random effects with Wald chi‐squared tests and likelihood ratio tests, respectively. Two individuals who were outliers for gall number were excluded from this analysis. Bold text indicates statistically significant terms at *α* = 0.05.

## 4 DISCUSSION

We found that the soil microbiome has a major impact on *M*. *lupulina* survival in the field. Plants were twice as likely to survive when grown in soil with an intact microbial community compared to microbiome‐depleted soil. The microbiome also modified belowground interactions with two root endosymbionts. Parasitic nematode infection was significantly greater in plants inoculated with an intact soil microbial community, although this effect may be simply attributed to additional viable nematode eggs in the live soil inoculum. The intact soil microbiome also modified plant interactions with rhizobia in a genotype‐specific manner, increasing nodulation in some genotypes and decreasing it in others. Our results suggest that an intact soil microbiome has complex effects on plants under natural conditions, increasing survival and reshaping belowground plant–symbiont interactions.

### 4.1 An intact microbiome increased plant survival but did not affect other plant traits

The consensus from previous research is that the soil microbial community is essential for plants (Busby et al., 2017; Chaparro et al., 2012). Our results are consistent with this finding, but our data illustrate just how dramatic this effect is under field conditions (Figure 3). In our experiment, *M*. *lupulina* plants grown in the intact microbiome treatment were twice as likely to survive as plants grown in microbiome‐depleted soil. Our data confirm that at least some of the benefits associated with an intact soil microbiome in the laboratory persist in the field (Berendsen et al., 2012; Porter et al., 2020), and are consistent with past work showing that symbionts help plant to withstand stressful biotic and abiotic environments (Griffiths et al., 2000; Porter et al., 2020; Rolli et al., 2015), which is likely disproportionately important in the field relative to the laboratory. Most mortality in our experiment occurred shortly after transplantation into the field mesocosms, which suggests that the microbiome may have provided protection against the suite of stresses associated with transplantation.

It is unlikely that the effect of the microbiome treatment on plant survival in our experiment is attributable to nitrogen‐fixing rhizobia because all plants in our experiment were inoculated with these mutualists. Instead, our data are consistent with other research that has found that while individual symbionts do play a role in plant growth, entire soil microbial communities have a major effect on plant health as well (Chaparro et al., 2012; Saleem et al., 2019). Because we did not sequence the soil microbial communities in our experiment, we do not know exactly how the communities differed between microbiome treatments (e.g., differences in the overall population size of microbes, community composition, or both?). Several underlying mechanisms may therefore be responsible for the patterns we observed. For example, plants grown in the intact microbiome treatment may have been more likely to survive due to the functional benefits arising from greater soil microbial species richness (Lau & Lennon, 2011; Wagg et al., 2019). It is worth noting that because the live soil used in our intact microbiome treatment was collected from wild *M*. *lupulina* plants, it was presumably enriched for *M*. *lupulina*‐associated symbionts, and a phenomenon is known as a plant–soil feedback (Bever, 1994; Putten et al., 2013, 2016). Enrichment of host‐associated symbionts is not always beneficial, because hosts inadvertently enrich for parasites and pathogens as well as mutualists (Bever, 1994; Putten et al., 2016), but our experiment demonstrates that the net effect of this *M*. *lupulina*‐associated community on plant survival was positive, that is, that the benefits derived from mutualists and commensals outweighed the harms imposed by parasites and pathogens. To determine whether the large effect of the rhizosphere microbiome on host survival is due to the presence of microbes in general, or to host‐associated microbes in particular, future experiments could compare host survival in soil communities sampled from the host rhizosphere to survival in soil from locations where the host is absent.

Finally, changes in soil properties caused by autoclaving may have contributed to mortality in the microbiome‐depleted treatment. Autoclaving is a standard method for sterilizing soil in plant studies (Berns et al., 2008; Petipas et al., 2021; Trevors, 1996), but it does change soil properties, increasing dissolved organic matter, destroying soil structure, and altering the concentrations of essential nutrients and elements that can be toxic for plants at high concentrations (Berns et al., 2008; Trevors, 1996). However, we think it is unlikely that the huge difference in mortality between microbiome treatments was an artifact of the sterilization procedure because other studies employing a similar approach (Petipas et al., 2020) do not report a large difference in plant mortality between sterilized and non‐sterilized soil treatments.

With such large differences in survival, we were surprised to see that the microbiome did not affect any other measure of host performance (shoot biomass, root biomass, or shoot‐to‐root ratio; Figure 4). Nor did we detect an effect of nematode infection on plant growth (Figure 4). The absence of these treatment effects on plant performance could be a by‐product of our experimental design. Greater differences in plant traits may have been apparent if our study had run longer, allowing us to capture effects on fitness components expressed later in life, such as the timing of reproduction or reproductive success (Gould et al., 2018; Lau & Lennon, 2012; Metcalf et al., 2019; Panke‐Buisse et al., 2015). It is also possible that differences between our two microbiome treatments attenuated over time as the microbiome‐depleted treatment was colonized by ambient microbes, weakening the effect of the microbiome treatment on traits such as biomass that were measured late in the experiment. High mortality in the microbiome‐depleted treatment also reduced our statistical power to detect treatment effects in later life stages.

Another potential explanation as to why there was no difference in biomass between microbiome treatments is that the rhizobia mutualism largely compensated for the negative impact of a depleted microbiome, given that all plants in our experiment were inoculated with these key mutualists. The strong positive relationship between the rhizobia mutualism and plant biomass is well established in the literature (Heath, 2010; Masson‐Boivin & Sachs, 2018; Wood et al., 2018). If this key mutualism did indeed shield the host from the negative impacts of a depleted rhizosphere microbiome, it indicates how important it is to experimentally disentangle the effect of major host‐microbe mutualisms from the effect of the microbiome as a whole on host performance. Without doing so, we may consistently overestimate the physiological importance of the microbiome as a whole, when in fact its benefits are tied to a small number of important taxa. Still, rhizobia are not the only microbe to promote plant growth: Others, such as mycorrhizal fungi and other root endophytes, also increase plant biomass (Afkhami & Stinchcombe, 2016; Bonfante & Genre, 2010; Hardoim et al., 2015), so the lack of difference in biomass between treatment groups of our experiment remains a surprising result.

Finally, the large effect of the rhizosphere microbiome on survival but not biomass may be a manifestation of the "invisible fraction" problem (Bennington & McGraw, 1995; Weis, 2018). This problem arises because early‐life mortality—in our experiment, mortality caused by our microbiome treatment—biases measurements of traits expressed later in life whenever individuals with different trait values differ in their probability of survival. The individuals that die are known as the "invisible fraction" because they are unmeasurable, and therefore cannot contribute to estimates of treatment effects that manifest after mortality occurs. The invisible fraction problem could account for the absence of a microbiome effect on plant biomass if the surviving plants in the microbe‐depleted treatment were more resilient to the negative phenotypic effects of a depleted microbiome (e.g., resistant to stress or more efficient at resource uptake) than those that died.

### 4.2 The microbiome modified plant interactions with two belowground endosymbionts

The microbiome treatment impacted colonization by both belowground symbionts in our experiment. It had a particularly large effect on nematode infection: Plants in the intact microbiome treatment formed many times more galls than those in the microbiome‐depleted treatment (Figure 5a). However, this effect may simply be due to viable nematode eggs in the live soil. Consistent with this is the fact that among plants that were not inoculated with nematodes, more than half the plants in the intact microbiome treatment formed galls, while only one plant in the microbiome‐depleted treatment formed any galls. Therefore, we cannot exclude the possibility that the intact microbiome treatment promoted nematode infection simply because the live soil contained nematode eggs.

Our result—that the microbiome either did not affect or increased host susceptibility to parasitic nematodes—conflicts with a previous experiment in the same group of parasites, which found that host‐associated rhizosphere microbes reduced infection by suppressing root invasion and reproduction of these nematodes (Elhady et al., 2018). In fact, most work to date on the role of the microbiome in infectious disease has found that host‐associated microbes tend to protect their host from parasites and pathogens (King & Bonsall, 2017; Vannier et al., 2019). In mammalian intestines, beneficial microbes interact with host immunity to hinder pathogen colonization, and experimentally disrupting the gut microbiome increases the risk of infection (Hernandez et al., 2019; Sassone‐Corsi & Raffatellu, 2015). This principle even extends to vectors of infectious disease: In these animals, the microbiome reduces vector competence, resulting in a concomitant decrease in disease transmission (Weiss & Aksoy, 2011). By contrast, we did not detect any protective effect of the microbiome in our experiment: If anything, our data indicate that the microbiome *increased* host susceptibility to parasite infection. One possible explanation is that parasites prefer hosts with intact microbiomes, perhaps because beneficial microbes increase the plant's nutritional value as a host. A similar phenomenon has been documented in aboveground herbivores (Chen et al., 2010). However, this hypothesis is not consistent with the lack of a difference in biomass between plants with intact and depleted microbiomes. Another possibility is that the soil microbial community modifies plant immune function, influencing the host's ability to filter the microorganisms invading its root system. Future research should explore the mechanisms by which the microbiome could increase a host's vulnerability to infectious disease.

To our surprise, parasitic nematodes did not impact plant performance in our experiment. Our nematode treatment did not affect survival nor any of the traits we measured, and gall number was not related to shoot biomass, a commonly used fitness proxy (Tables 1, 2, 3). One explanation for the absence of a cost of parasitism in our experiment is that nematodes affect other fitness proxies. In a greenhouse study in *Medicago truncatula*, *Meloidogyne* nematodes decreased fruit production but not shoot biomass (Wood et al., 2018). At comparable inoculation densities to what we used in the present study, *Meloidogyne* nematodes also significantly decreased yields of several crops in the field, including tomato and soybean (Barker et al., 1976).

The microbiome also affected the rhizobia mutualism, although its effect depended strongly on plant genotype (genotype‐by‐microbiome interaction; Table 2, Figure 6d). An intact microbiome increased nodulation in some plant genotypes and decreased it in others. Our results suggest that the host plant mediates how the broader soil microbial community interacts with rhizobia, either directly or indirectly. Such context dependence has emerged as a characteristic feature of the legume‐rhizobia mutualism (it has previously been reported in other environmental variables, e.g., nitrogen), and it is hypothesized to contribute to the maintenance of genetic variation in this ecological significant symbiosis (Heath & Tiffin, 2007).

There are several possible mechanisms underlying the plant genotype‐dependent impact of the microbiome on nodulation in our experiment. First, in our experiment, plants in the intact microbiome treatment almost certainly were exposed to resident rhizobia in the live soil inoculum in addition to the rhizobia strain we inoculated onto all plants, while plants in the microbiome‐depleted treatment only received the strain we inoculated. As a result, plants in the intact microbiome treatment likely received a higher dose of rhizobia and a more diverse population of rhizobia strains. Because the most beneficial rhizobia strain often differs among plant genotypes, plants in the intact microbiome treatment may have benefited from the ability to choose the most beneficial strain (Heath & Tiffin, 2007; Pahua et al., 2018).

Alternatively, it is possible that communication between rhizobia and other bacteria in the soil (i.e., quorum sensing) may underlie the increase in nodulation (Miao et al., 2018). It has also recently become clear that rhizobia are not the only bacteria living in nodules (Martínez‐Hidalgo & Hirsch, 2017; Tapia‐García et al., 2020). If these co‐habiting bacteria facilitate nodulation, it could account for the effect of the microbiome on nodulation we observed, especially if plant genotypes vary in their recruitment of these nodule‐associated bacteria. A second possibility is that the rhizosphere microbiome primes the plant immune response, influencing subsequent interactions with symbionts, including rhizobia (Cameron et al., 2013; Nishad et al., 2020; Pršić & Ongena, 2020). This phenomenon, known as systemic acquired resistance, could account for the variable impact of the microbiome on nodulation in our experiment if plant genotypes vary in the degree to which their immune system is primed by early‐life encounters with symbionts. One weakness of this hypothesis, however, is that it does not explain why the microbiome only impacted nodulation by rhizobia and not gall formation by nematodes. Finally, the impact of the rhizosphere microbiome on nodulation could be mediated by nitrogen availability. Although rhizobia are unique in their ability to engage in symbiotic nitrogen fixation, other free‐living soil prokaryotes known as diazotrophs are also capable of converting nitrogen gas into biologically available forms (biological nitrogen fixation; Bueno Batista & Dixon, 2019; Nag et al., 2020). If plant genotypes vary in the extent to which they recruit these diazotrophs, the resulting differences among genotypes in soil nitrogen availability could explain why some genotypes decreased investment in the rhizobial mutualism when the microbiome was present. Recent work in *Medicago* has found that rhizosphere community composition is shaped by plant genotype, consistent with this hypothesis (Brown et al., 2020).

## 5 CONCLUSIONS

Here, we showed that the rhizosphere microbiome has large and wide‐ranging effects on *Medicago lupulina* plants growing in the field. Our experiment is notable in that it demonstrates that these microbiome‐mediated effects—well established under controlled laboratory conditions—persist under ecologically realistic conditions. Future studies should explore the mechanisms that underlie the patterns we observed, to shed light on the functional relationships between plants and the symbionts that they host. Additionally, researchers should examine when and how the microbiome acts as a major agent of selection on plant traits—especially traits mediating host–symbiont interactions—to better understand the evolutionary consequences of these microscopic ecological communities.

## CONFLICT OF INTEREST

We have no conflict of interest to disclose.

## AUTHOR CONTRIBUTION

**Shaniya Markalanda:** Conceptualization (equal); Investigation (equal); Methodology (equal); Writing‐original draft (lead). **Connor McFadden:** Conceptualization (equal); Investigation (equal); Methodology (equal); Writing‐review & editing (supporting). **Steven Cassidy:** Project administration (equal); Supervision (equal); Writing‐review & editing (supporting). **Corlett Wolfe Wood:** Conceptualization (equal); Formal analysis (lead); Funding acquisition (lead); Project administration (equal); Supervision (equal); Writing‐review & editing (lead).

### OPEN RESEARCH BADGES

This article has earned an Open Data Badge for making publicly available the digitally‐shareable data necessary to reproduce the reported results. The data is available at https://doi.org/10.5061/dryad.76hdr7sxs

## Data Availability

The data and associated R code are available on Dryad: https://doi.org/10.5061/dryad.76hdr7sxs
